# Großbefragungen zur Arbeitssituation von Menschen mit gesundheitlicher Beeinträchtigung und Behinderung in Deutschland – Erweiterung des Analysepotenzials

**DOI:** 10.1007/s00103-026-04202-0

**Published:** 2026-03-04

**Authors:** Lena Hünefeld, Alper Eker, Sophie Teborg

**Affiliations:** Bundesanstalt für Arbeitsschutz und Arbeitsmedizin, Friedrich-Henkel Weg 1–25, 44149 Dortmund, Deutschland

**Keywords:** Befragungen, Berufliche Teilhabe, Menschen mit Behinderung, Inklusion, Arbeitsbedingungen, Surveys, Participation in working life, People with disabilities, Inclusion, Working conditions

## Abstract

**Einleitung:**

Eine fundierte und inklusive Gestaltung von Arbeitsplätzen erfordert eingehendes Wissen über die Arbeitssituation – erfasst durch geeignete, aktuelle Indikatoren. Befragungsdaten ermöglichen eine differenzierte Beschreibung dieser Situation und das Nachvollziehen von Entwicklungen im Zeitverlauf. Ziel dieses Beitrags ist eine systematische Übersicht über Großbefragungen zur Arbeitssituation von Menschen mit Beeinträchtigung und Behinderung in Deutschland sowie eine Untersuchung des Analysepotenzials.

**Methoden:**

Insgesamt 15 Großbefragungen, die eine Identifizierung von Personen mit Beeinträchtigung ermöglichen und Informationen zur Arbeitssituation liefern, wurden systematisch analysiert. Dabei wurden die Befragungen anhand eines induktiv und deduktiv entwickelten Kategoriensystems betrachtet, das die Themen „Beeinträchtigung/Behinderung“, „Arbeitssituation“ und „individuelle Merkmale“ abdeckt. Ferner wurde unterschieden, ob die Befragungen die Zielgruppen „Menschen mit Beeinträchtigung“, „Menschen mit selbsteingeschätzter Behinderung“ und/oder „Menschen mit fremdeingeschätzter Behinderung“ einbeziehen.

**Ergebnisse:**

Für den Zeitraum 2018 bis 2024 lassen sich 7 relevante Befragungen identifizieren, die in unterschiedlichem Ausmaß differenzierte Informationen zu allen 3 Zielgruppen und ihrer Arbeitssituation auf dem ersten Arbeitsmarkt enthalten.

**Diskussion:**

Deutlich werden Lücken in den Befragungsdaten, insbesondere im Hinblick auf inklusive betriebliche Rahmenbedingungen oder Arbeitsplatzanpassungen. Bestehende Großerhebungen müssten daher systematisch erweitert werden – inhaltlich durch differenzierte Items, aber auch methodisch durch inklusive Stichproben und Erhebungsdesigns.

**Zusatzmaterial online:**

Zusätzliche Informationen sind in der Online-Version dieses Artikels (10.1007/s00103-026-04202-0) enthalten.

## Abstract (en)

**Introduction:**

Sound knowledge of the current work situation, captured using suitable and up-to-date indicators, is a key prerequisite for the appropriate and accessible design of working conditions. Survey data enable a differentiated description of this situation and the tracking of developments. The aim of this article is to provide a systematic overview of large-scale surveys on the work situation of people with impairments or disabilities in Germany and to examine the potential for analysis.

**Methods:**

A total of 15 large-scale surveys involving people with health impairments and providing information on their work situation were systematically analyzed. An inductively and deductively developed category system was used that structured the topics as “impairment/disability,” “work situation,” and “individual characteristics.” In addition, a distinction was made between three target groups: “people with impairments,” “people with self-assed disabilities,” and/or “people with externally assessed disabilities.”

**Results:**

For the period from 2018 to 2024, seven relevant surveys can be identified that, to varying extents, contain detailed information on all three target groups and their employment situation in the primary labor market.

**Discussion:**

Gaps in the survey data become apparent, for example, with regard to inclusive working conditions or workplace adaptations. Therefore, existing large-scale surveys would need to be systematically expanded in terms of both content, through differentiated items, and methodology, through inclusive sampling and survey designs.

## Einleitung

In Deutschland leben etwa 7,9 Mio. Menschen mit einer Schwerbehinderung, die 9,3 % der Gesamtbevölkerung ausmachen. Des Weiteren sind 3,1 Mio. Menschen mit Schwerbehinderung im erwerbsfähigen Alter von 15 bis 64 Jahren [1]. Die gleichberechtigte Teilhabe am Arbeitsleben ist ein zentrales Element gesellschaftlicher Inklusion und ökonomischer Unabhängigkeit. Für Menschen mit einer gesundheitlichen Beeinträchtigung bzw. einer Behinderung ist die Realisierung dieses Rechts jedoch nach wie vor mit besonderen Herausforderungen verbunden. In Deutschland zeigen sich trotz gesetzlicher Vorgaben und vielfältiger Inklusionsbemühungen weiterhin strukturelle Benachteiligungen beim Zugang zu Arbeit, bei den Arbeitsbedingungen sowie hinsichtlich beruflicher Entwicklungsmöglichkeiten auf dem ersten Arbeitsmarkt [2–5]. Diese Ungleichheiten manifestieren sich sowohl in einer niedrigeren Erwerbstätigenquote von Menschen mit einer Schwerbehinderung (51,4 % vs. 79,3 %; [6]) als auch in Aspekten wie Arbeitszufriedenheit, Einkommen oder Passung von Arbeitsanforderungen und individuellen Fähigkeiten [3, 7, 8].

Damit Arbeit zur Förderung der Gesundheit beitragen kann, muss sie den Menschen in den Mittelpunkt stellen und sich an seinen Bedürfnissen orientieren [9]. Die Arbeitsbedingungen müssen dazu unter anderem so gestaltet sein, dass Autonomie, Teilhabe und Selbstwirksamkeit ermöglicht werden [10]. Für Menschen mit Beeinträchtigung bzw. Behinderung umfasst dies etwa den Abbau physischer, kommunikativer und struktureller Barrieren. Ein barrierefreier Arbeitsplatz, der auch soziale und organisatorische Rahmenbedingungen einschließt, ist eine wesentliche Voraussetzung für gleichberechtigte Teilhabe [11–13]. Ebenso zentral ist, dass arbeitsbezogene Belastungen individuell tragbar gestaltet und Unterstützungsbedarfe frühzeitig erkannt werden [13–15].

Die UN-Behindertenrechtskonvention (UN-BRK) sichert Menschen mit Behinderung das Recht auf Arbeit und auf gerechte Arbeitsbedingungen, Chancengleichheit, gleichen Lohn, sichere Arbeitsbedingungen und Schutz vor Missständen zu [16]. Um die Umsetzung dieser Rechte in Deutschland zu beurteilen, müssen geeignete Informationen, einschließlich statistischer Angaben und Forschungsdaten, gesammelt und analysiert werden [16]. Viele der datenbezogenen Informationen über Menschen mit Beeinträchtigung bzw. Behinderung basieren auf amtlichen Statistiken oder Routinedaten^1^ [17]. Dabei liegt der Fokus überwiegend auf Menschen, „… deren Beeinträchtigung oder Behinderung behördlich anerkannt ist und/oder die öffentliche (Sozial‑)Leistungen beziehen“ [4]. Daten über Personen ohne Leistungsbezug werden jedoch meist nicht erfasst.

Befragungsdaten stellen eine sehr gute Ergänzung dar, um nicht nur Informationen über Menschen mit einer amtlich anerkannten Behinderung bzw. Schwerbehinderung zu erhalten, sondern auch über Personen mit länger andauernden Erkrankungen oder gesundheitlichen Beeinträchtigungen. Des Weiteren stellen Befragungen die Perspektiven der Befragten selbst in den Mittelpunkt. Dies ermöglicht, die Arbeitssituation von Menschen mit Beeinträchtigung bzw. Behinderung differenziert zu beschreiben und Entwicklungen im Zeitverlauf nachzuvollziehen [18]. Sie liefern wichtige Hinweise über Faktoren, die besondere Aufmerksamkeit verdienen, wie Merkmale von Betroffenen oder Kontextfaktoren von potenziellen Benachteiligungen, und ermöglichen eine datenbasierte Steuerung politischer Maßnahmen.

Vor diesem Hintergrund verfolgt der Beitrag das Ziel, einen systematischen Überblick über relevante Großbefragungsdaten in Deutschland zu geben, die Aussagen zur Arbeitssituation von Menschen mit Beeinträchtigung bzw. Behinderung auf dem ersten Arbeitsmarkt ermöglichen. Dabei werden sowohl die Potenziale der bestehenden Datengrundlage herausgearbeitet als auch bestehende Lücken identifiziert. Diese Übersicht bietet Forschenden eine Orientierung und gibt politischen und institutionellen Akteur*innen Impulse für die Weiterentwicklung der Erhebungslandschaft, um langfristig eine inklusive Arbeitswelt datenbasiert zu gestalten.

## Methoden

Das methodische Vorgehen gliedert sich in 3 zentrale Schritte: (1) Identifizierung relevanter Großbefragungen, (2) die Auswahl geeigneter Erhebungen auf Basis definierter Ein- und Ausschlusskriterien sowie (3) die inhaltliche Analyse mittels eines eigens entwickelten Kategoriensystems.

### Identifikation relevanter Großbefragungen

Die Identifikation potenziell geeigneter Befragungen erfolgte zum einen durch eine strukturierte Recherche in den Datenbeständen ausgewählter Forschungsdatenzentren (FDZ), wie zum Beispiel des Instituts für Arbeitsmarkt- und Berufsforschung (IAB), des statistischen Bundesamtes und GESIS - Leibniz-Institut für Sozialwissenschaften. Zum anderen wurden einschlägige wissenschaftliche Publikationen [z. B. 19–22] und Berichte [z. B. 3, 23, 24] analysiert. Dabei wurden sowohl Befragungen berücksichtigt, die sich spezifisch nur auf erwerbstätige Personen beziehen, als auch Befragungen, die Personen in Privathaushalten als Zielpersonen hatten. Mit Großbefragungen sind hier Befragungen gemeint, die mindestens 1000 Personen erfassen und repräsentative (Teil‑)Stichproben untersuchen. Eine Übersicht über die 36 identifizierten Befragungen befindet sich in Tabelle Z1 im Onlinematerial.

### Auswahlkriterien der einbezogenen Befragungen

Die Auswahl der in die Analyse einbezogenen Befragungen erfolgte auf Grundlage zuvor definierter Ein- und Ausschlusskriterien. Die Mindestanforderung an die Befragungen war, dass Menschen mit andauernden gesundheitlichen Beeinträchtigungen identifizierbar sind. Im Idealfall boten die Befragungen darüber hinaus Informationen zur Gruppe der Menschen mit Behinderung, indem sie Informationen zu Teilhabeeinschränkungen (selbsteingeschätzte Behinderung) oder zu einer amtlichen Anerkennung der Behinderung (fremdeingeschätzte Behinderung) enthielten. Darüber hinaus musste in der Befragung die Zielgruppe von erwerbstätigen Personen auf dem ersten Arbeitsmarkt (mit) abgedeckt und mindestens eine Information zur Arbeitssituation enthalten sein (z. B. berufliche Tätigkeit, betriebliche Rahmenbedingungen oder Arbeitsbedingungen). Insgesamt mussten die Befragungen die folgenden 6 Kriterien erfüllen, um berücksichtigt zu werden:

1. Vorhandensein von Variablen zur Identifikation einer andauernden gesundheitlichen Beeinträchtigung,
2. (Teil‑)Stichprobe von Erwerbstätigen auf dem 1. Arbeitsmarkt und Abdecken des Themengebiets Arbeit,
3. repräsentativ für die deutsche (Erwerbs‑)Bevölkerung oder relevante Teilpopulationen,
4. bei europäischen Erhebungen das Vorliegen einer deutschen Teilstichprobe,
5. Zugänglichkeit für interessierte Wissenschaftler*innen (z. B. über FDZ oder Open-Access-Repositorien; Stand: Mai 2024),
6. Erhebungszeitraum zwischen 2018 und 2024, um eine möglichst aktuelle Datenbasis zu gewährleisten.

### Entwicklung des Kategoriensystems

Die systematische inhaltliche Erfassung der ausgewählten Befragungen erfolgte mithilfe eines iterativ entwickelten Kategoriensystems. Dieses wurde in mehreren Schritten überarbeitet, um die Heterogenität der Erhebungen angemessen abzubilden. In einem ersten Schritt wurden 3 thematische Bereiche definiert, anhand derer die Befragungen gesichtet wurden: 1. Beeinträchtigung/Behinderung, 2. Arbeitssituation, 3. individuelle Merkmale.

In einem zweiten Schritt wurden innerhalb der einzelnen Themenbereiche deduktiv und induktiv Haupt- und Unterkategorien entwickelt. Ausgehend vom differenzierten Verständnis von Beeinträchtigung und Behinderung der Teilhabeberichterstattung der Bundesregierung in Anlehnung an die „Internationale Klassifikation der Funktionsfähigkeit, Behinderung und Gesundheit“ (International Classification of Functioning, Disability and Health – ICF; [3, 25, 26]) sowie auf Basis des Operationalisierungskonzepts der „Repräsentativbefragung zur Teilhabe von Menschen mit Behinderungen“ des Bundesministeriums für Arbeit und Soziales (BMAS; [4]), wurde im Themenbereich „Beeinträchtigung/Behinderung“ die Hauptkategorie „selbst- und fremdeingeschätzte Beeinträchtigung bzw. Behinderung“ aufgenommen. Zu Menschen mit Beeinträchtigungen zählen Personen mit langfristigen Einschränkungen der Beweglichkeit, der Sinneswahrnehmung (Sehen, Hören, Sprechen), der psychischen Gesundheit oder der kognitiven Leistungsfähigkeit. Selbsteingeschätzte Behinderung ergibt sich aus der Kombination des subjektiv eingeschätzten Grads der gesundheitlichen Beeinträchtigung und der subjektiven Einschränkung im Alltag [27]. Die fremdeingeschätzte Behinderung beruht auf Informationen im Kontext einer amtlich anerkannten Behinderung. Liegen Informationen in einer Befragung sowohl zur gesundheitlichen Beeinträchtigung und zur Alltagseinschränkung als auch Informationen zu einer amtlich anerkannten Behinderung – inklusive des Grads der Behinderung – vor, können die folgenden Gruppen in einer Befragung unterschieden werden:

- *Menschen mit Beeinträchtigung:* wenn eine (mindestens 6 Monate) andauernde gesundheitliche Beeinträchtigung vorliegt,
- *Menschen mit selbsteingeschätzter Behinderung: *wenn mindestens eine andauernde gesundheitliche Beeinträchtigung vorliegt und das alltägliche Handeln nach eigener Einschätzung entweder deutlich oder stark eingeschränkt ist – oder wenn eine ausgeprägte Beeinträchtigung besteht, die den Alltag zumindest in gewissem Maße eingeschränkt [4],
- *Menschen mit fremdeingeschätzter (Schwer‑)Behinderung:* wenn eine sozialrechtlich anerkannte Behinderung nach SGB IX vorliegt. Bei Personen mit einer anerkannten Schwerbehinderung muss ein Grad von Behinderung von mindestens 50 vorliegen (§ 2 Abs. 2 SGB IX-neu; [27, 28]).

Die Entwicklung der Haupt- und Unterkategorien der Themenbereiche „Arbeitssituation“ und „individuelle Merkmale“ orientierte sich an vergleichbaren Übersichtsarbeiten im Bereich der Arbeits- und Gesundheitsforschung [20, 21] und wurde in Anlehnung an bestehende Forschungserkenntnisse zu inklusionsförderlichen Bedingungen [8, 13, 29] durch inklusionsspezifische Kategorien wie „Vorhandensein einer/eines Inklusionsbeauftragten“, „Umgang mit behinderten/chronisch kranken Menschen“ oder „barrierefreie Arbeitsbedingungen“ ergänzt. Das abschließende Kategoriensystem umfasst 14 Hauptkategorien und 112 Unterkategorien (Tab. 1 und 2). Anhand des Kategoriensystems wurden in einem abschließenden Schritt alle eingeschlossenen Befragungen gesichtet und die Anzahl der Items pro Unterkategorie sowie die Variablennummer in einer Übersichtstabelle festgehalten (siehe Tabelle Z2 und Z3 im Onlinematerial). Es wurde jeweils die aktuellste vorliegende Welle berücksichtigt, die im Mai 2024 verfügbar war.

**Tab. 1:** Erfasste Kategorien und Unterkategorien in den thematischen Bereichen Beeinträchtigung/Behinderung, Arbeitssituation und individuelle Merkmale

Hauptkategorien	Unterkategorien
*Beeinträchtigung/Behinderung*	
Selbsteingeschätzte gesundh. Beeinträchtigung/Behinderung	Gesundheitsproblem/Beschwerden (chronisch, überdauernd), Alter/Zeitpunkt des Auftretens der Gesundheitsprobleme/Beschwerden, weitere Gesundheitsinformationen (z. B. allgemeiner Gesundheitszustand, Beschwerden), Art der Beeinträchtigung, Stärke der Beeinträchtigung, Hilfsmittelnutzung im Alltag, Hilfen im Alltag (z. B. durch andere Personen, Leistungen Pflegeversicherung), Alltagseinschränkungen, Arbeitseinschränkungen, Arbeitsfähigkeit
Fremdeingeschätzte Behinderung	Amtlich anerkannte Behinderung/Erwerbsminderung, Jahr der Feststellung/Anerkennung, Grad der Behinderung (GdB)
*Arbeitssituation*	
Stellung im Arbeitsmarkt	Beruf/berufliche Tätigkeit, Stellung im Beruf, Anforderungsniveau der Tätigkeit, Führungsverantwortung/Projektleitung, Befristung, Zeitarbeit, geringfügige Beschäftigung, Arbeitslosigkeitserfahrung, finanzielle Situation der Person/des Haushaltes, Erwerbsbiografie, Passung Ausbildung – Tätigkeit, Nebentätigkeit, Dauer Betriebszugehörigkeit
Unternehmensmerkmale	Branchenzugehörigkeit, Betriebsgröße, Umstrukturierungen, Mitarbeiterstruktur, öffentlicher Dienst/Privatunternehmen, wirtschaftliche Lage des Betriebes
Betriebliche Rahmenbedingungen	Personal‑/Betriebsrat/Gewerkschaft vorhanden, Beauftragte/r für Arbeitsschutz und Gesundheit vorhanden, Inklusionsbeauftragte/r vorhanden, Arbeitszeiterfassung/-modell/-regelung, Fort- und Weiterbildungsmöglichkeiten, Vereinbarkeit Familie und Beruf, individuelle Gestaltungsmöglichkeiten/Einflussnahme, Umgang mit behinderten/chronisch kranken Menschen, Unternehmenskultur/Betriebsklima, soziale Verantwortung, nichtmonetäre Zusatzleistungen, Personalengpässe
Rahmenbedingungen der Tätigkeit	Homeoffice/Telearbeit, Pendelweg/Arbeitsweg, mobiles Arbeiten/Arbeitsort, Arbeit mit Technik/Werkzeugen/digitalen Medien, (Ausmaß) Kundenorientierung, Dienstreisen, Ergebnisorientierung/Eigenverantwortung, Zielvereinbarungen, Leistungssteuerung/-kontrolle, sozialer Kontext (z. B. Projekt‑/Teamarbeit, Abhängigkeit von Kolleg*innen), Karrieremöglichkeiten
Arbeitszeitmerkmale	Arbeitszeitlänge, Arbeitszeitwünsche, Bereitschaftsdienst, Rufbereitschaft, Arbeit auf Abruf, Pausen, Überstunden, flexible Arbeitszeitmodelle, Nacht‑/Schichtarbeit, Wochenendarbeit, Einfluss auf Arbeitszeit, Änderungen Arbeitszeiten, ständige Erreichbarkeit
Arbeitsbedingungen	Körperliche Belastungen, umgebungsbezogene Belastungen, psychosoziale Belastungen, barrierefreie Arbeitsbedingungen, Hilfsmittel im Arbeitskontext (Tab. 2)
Arbeitszufriedenheit und Arbeitsmotivation	Allgemeine Arbeitszufriedenheit, Zufriedenheit mit einzelnen arbeitsbezogenen Merkmalen, Arbeitsmotivation
*Individuelle Merkmale*	
Individuelle Merkmale	Geschlecht, Alter, Schul‑/Ausbildungsabschluss, Familienstatus, Migrationshintergrund/-erfahrung, soziale Unterstützung privates Umfeld, Karriereorientierung, Mitglied Gewerkschaft

**Tab. 2:** Erfasste Unterkategorien für Arbeitsbedingungen

Unterkategorien 1	Unterkategorien 2
Körperliche Belastungen	Heben/Tragen, Körperhaltungen, wiederholte Bewegungsabläufe, physisches Arbeitsaktivitätsniveau
Umgebungsbezogene Belastungen	Umgang Mikroorganismen/Gefahrstoffe, Hitze/Kälte/Rauch/Staub etc., Lärm, Beleuchtung, Vibrationen, Schutzkleidung
Psychosoziale Belastungen	Arbeitsmenge/-tempo, emotionale Anforderungen, Lern-/kognitive Anforderungen, Über- oder Unterforderung, Sinnhaftigkeit/wichtige Tätigkeit, widersprüchliche Anforderungen, Einflussmöglichkeiten, Handlungsspielräume/Freiheitsgrade, Abwechslung/Monotonie, Arbeitsunterbrechungen, Führungsstil/Führungsqualität, Vorhersagbarkeit/Planbarkeit, Wertschätzung/Fairness, Unterstützung durch Vorgesetzte, Unterstützung durch Kolleg*innen, übergriffiges Verhalten/Belästigung/Diskriminierung, Arbeitsplatz(un)sicherheit, arbeitsbezogene Konflikte
Barrierefreie Arbeitsbedingungen	–
Hilfsmittel im Arbeitskontext	–

## Ergebnisse

Von den anfänglich identifizierten 36 Befragungen erfüllten 15 Befragungen alle 6 Einschlusskriterien (Tab. 3).

**Tab. 3:** Übersicht über die eingeschlossenen Befragungen

Nr.	Befragungen	Datenzugang über	Startdatum	Aktuellste Welle	Erhebungsdesign	Kernthemen
1	Aufwachsen in Deutschland: Alltagswelten (AID:A)	Deutsches Jugendinstitut (DJI)	2009	2019	Längsschnitt: Hauptwellen 2009, 2014, 2019; Zwischenbefragungen 2012–2014, 2014–2016, 2020, 2021	Lebenszufriedenheit, Corona-Folgen, Freizeit, Politik, Selbstbild, Schule/Arbeit, Beziehungen, Familie
					CAPI, CATI	
					Zielgruppe: Personen 0–32 Jahre in Privathaushalten	
2	BIBB/BAuA-Erwerbstätigenbefragung (BIBB/BAuA)*	Bundesinstitut für Berufsbildung (BIBB)	1979 (vergleichbar ab 2006)	2024	Querschnitt: alle 5–6 Jahre	Arbeitsbedingungen, Arbeitsbelastung, Ausbildung, Gesundheit
					CATI	
					Zielgruppe: Erwerbstätige ab 15 Jahren mit ≥ 10 h entlohnter Arbeit/Woche	
3	BIBB-Nachbefragung Menschen mit Behinderung 2019 (BIBB/BAuA-Zusatz)*	Bundesinstitut für Berufsbildung (BIBB)	2019	2019	Querschnitt: einmalig	Berufliche Teilhabe, Behinderung, Hilfsmittel, Gesundheit, Erwerbstätigkeit
					CATI	
					Zielgruppe: Erwerbstätige ab 15 Jahren mit und ohne Behinderung, ≥ 10 h Arbeit/Woche	
4	Deutscher Alterssurvey (DEAS)*	Deutsches Zentrum für Altersfragen (DZA)	1996	2020/2021	Längsschnitt: kohortensequenziell alle 3–6 Jahre	Lebenssituationen und Alternsverläufe
					CAPI; 2020 CAPI-by-phone; Drop-off CAWI, PAPI (selbst ausgefüllt)	
					Zielgruppe: Personen 40–85 Jahre in Privathaushalten	
5	European Social Survey: Germany (ESS)	GESIS (für Deutschland)	2002 (Deutschland)	2020/2021	Querschnitt: alle 2 Jahre	Gesellschaftliche Strukturen, Lebensumstände, Einstellungen
					CAPI, PAPI	
					Zielgruppe: Personen ab 15 Jahren in Privathaushalten in Europa	
6	European Union Statistics on Income and Living Conditions (EU-SILC)	Statistisches Bundesamt (Destatis)	2005	2021/2022	Querschnitt und Längsschnitt: jährlich	Einkommen, Armut, soziale Ausgrenzung und Lebensbedingungen
					PAPI, CAWI, CATI, Websurvey	
					Zielgruppe: Personen ab 16 Jahren in Privathaushalten	
7	European Working Conditions Survey (EWCS)	Eurofound	1990	2020	Querschnitt: ca. alle 5 Jahre	Arbeitsbedingungen, Gesundheit
					CATI, zuvor CAPI	
					Zielgruppe: Erwerbstätige ≥ 16 Jahre, ≥ 1 h/Woche, entlohnt	
8	Gesundheit in Deutschland aktuell (GEDA)	Robert Koch-Institut (RKI)	2008/2009	2019/2020	Querschnitt: 2009, 2010, 2012, 2014/2015	Gesundheit und Lebenssituation
					CATI	
					Zielgruppe: Personen ab 15 Jahren in Privathaushalten	
9	International Social Survey Programme (ISSP)	ISSP Research Group (für Deutschland: GESIS)	1984	2021	Querschnitt: jährlich	Wechselnde Schwerpunkte, 2021: Gesundheit und Gesundheitsfürsorge
					CAWI, CAPI, CATI, PAPI, PAPI (selbst ausgefüllt)	
					Zielgruppe: Personen ab 18 Jahren	
10	Leben in der Arbeit (lidA)*	Bergische Universität Wuppertal	2011	2021	Längsschnitt: kohortensequenziell alle 3–4 Jahre	Gesundheit und Älterwerden in der Arbeit
					CAPI und CAPI-by-phone	
					Zielgruppe: ehemals sozialversicherungspflichtig Beschäftigte der Jahrgänge 1959, 1965, 1971	
11	Mikrozensus (MZ)	Statistisches Bundesamt (Destatis)	1957 (gesamt ab 1991)	2021	Querschnitt: jährlich	Bevölkerungsstruktur, soziale Lage, Arbeitsmarkt, Ausbildung, Einkommen
					PAPI, CAWI, CATI	
					Zielgruppe: Personen ab 16 Jahren in Privathaushalten	
12	Panel Arbeitsmarkt und soziale Sicherung (PASS)*	Institut für Arbeitsmarkt- und Berufsforschung (IAB)	2006	2022	Längsschnitt: jährlich	Beschäftigung und Arbeitslosigkeit
					CATI, CAPI	
					Zielgruppe: Personen ab 15 Jahren in SGB-II- und Kontrollhaushalten	
13	Survey of Health, Ageing and Retirement in Europe (SHARE)	MEA am Max-Planck-Institut für Sozialrecht und Sozialpolitik	2004	2021/2022	Längsschnitt: alle 2 Jahre	Wirtschaftliche, gesundheitliche und soziale Lage älterer Menschen
					CAPI, Drop-off PAPI (selbst ausgefüllt)	
					Zielgruppe: Personen ab 50 Jahren in Privathaushalten und Einrichtungen	
14	Sozio-oekonomisches Panel (SOEP)*	Deutsches Institut für Wirtschaftsforschung (DIW)	1984	2021	Längsschnitt: jährlich	Einkommen, Erwerbstätigkeit, Bildung, Gesundheit, Lebenserwartung
					CAPI, PAPI	
					Zielgruppe: Personen in Privathaushalten	
15	Repräsentativbefragung zur Teilhabe von Menschen mit Behinderungen (Teilhabesurvey)*	Bundesanstalt für Arbeitsschutz und Arbeitsmedizin (BAuA)	2017	2023/2024	Längsschnitt: 2 Wellen, 2017–2021, 2023–2024	Teilhabe in allen Lebensbereichen
					CATI, CAPI, CASI, CAWI, PAPI	
					Zielgruppe: Menschen mit/ohne Beeinträchtigungen/Behinderungen ab 16 Jahren	

*CAPI* Computer Assisted Personal Interviewing; *CASI* Computer Assisted Self-Administered Interviewing; *CATI* Computer Assisted Telephone Interviewing; *CAWI* Computer Assisted Web Interview; *PAPI* Paper And Pencil Interviews

* Personen mit Beeinträchtigung, Personen mit selbsteingeschätzter Behinderung und Personen mit fremdeingeschätzter Behinderung identifizierbar

Mit Blick auf das Studiendesign der Befragungen wird deutlich, dass die Hälfte der Befragungen im Längsschnitt angelegt ist und Auswertungen von intraindividuellen Veränderungen und die Analyse von Langzeiteffekten prinzipiell ermöglicht. Hinsichtlich der Erhebungsmethoden zeigt sich ein breites Portfolio. Einige Befragungen nutzen ausschließlich eine (Haupt‑)Erhebungsmethode, wie zum Beispiel computergestützte Telefoninterviews (CATI; z. B. BIBB/BAuA; EWCS, GEDA). Andere Befragungen, wie ISSP, MZ oder Teilhabesurvey, bieten mehrere Erhebungsmethoden an, von Paper And Pencil Interviews (PAPI) über CATI bis hin zu Computer Assisted Web Interview (CAWI).

Hinsichtlich der Identifizierung der verschiedenen Zielgruppen (Menschen mit Beeinträchtigung, Menschen mit selbsteingeschätzter Behinderung und Menschen mit fremdeingeschätzter (Schwer‑)Behinderung) haben die eingeschlossenen Befragungen unterschiedliche Potenziale. Wie im Kapitel „Entwicklung des Kategoriensystems“ beschrieben, gilt hier das Operationalisierungskonzept im Teilhabesurvey als Referenz, da dort alle 3 Gruppen anhand einer Vielzahl von Items – insgesamt 260 Items zu selbst-/fremdeingeschätzter Beeinträchtigung/Behinderung – identifiziert und charakterisiert werden können (Tab. 4). Neben der Frage nach einer dauerhaften Beeinträchtigung und der Beeinträchtigung der körperlichen und psychischen Funktionen wird nach der Stärke der Beeinträchtigung bei Aktivitäten im Alltag, einer amtlich anerkannten Behinderung, dem Grad der Behinderung und dem Vorliegen eines Schwerbehindertenausweises gefragt. Darüber hinaus gibt es detaillierte Informationen zur Beeinträchtigungsart, der Hilfsmittelnutzung im Alltag und der Sichtbarkeit der Beeinträchtigung. In den Surveys EWCS und AID:A liegen lediglich Items vor, um Menschen mit Beeinträchtigungen zu identifizieren. In den Befragungen ESS, EU-SILC, GEDA, ISSP, und SHARE können zusätzlich Menschen mit selbsteingeschätzter Behinderung anhand der Informationen, ob eine andauernde gesundheitliche Beeinträchtigung vorliegt, und zum Grad der Einschränkung im alltäglichen Handeln identifiziert werden. Im MZ lassen sich wiederum Menschen mit Beeinträchtigung und fremdeingeschätzter (Schwer‑)Behinderung differenzieren. BIBB/BAuA, BIBB/BAuA-Zusatz, DEAS, lidA, PASS und SOEP lassen wie im Teilhabesurvey eine Differenzierung aller 3 Gruppen zu. Darüber hinaus bieten die verschiedenen Befragungen unterschiedliche Potenziale, gesundheitliche Beschwerden, die Art der Beeinträchtigung oder Alltags- und Arbeitseinschränkungen zu beleuchten, welches auf verschiedene Schwerpunkte der Befragungen zurückzuführen ist. So bieten zum Beispiel SHARE und GEDA – beides Befragungen mit einem Schwerpunkt auf Gesundheit – mit insgesamt 151 bzw. 103 Items eine Vielzahl an Möglichkeiten, die gesundheitliche Gesamtsituation zu beleuchten (Tab. 4).

**Tab. 4:** Untersuchte Befragungen – Anzahl der erfassten Items in den jeweiligen Hauptkategorien

	AID:A	BIBB/BAuA	BIBB/BAuA-Zusatz	DEAS	ESS	EU-SILC	EWCS	GEDA	ISSP	lidA	MZ	PASS	SHARE	SOEP	Teilhabesurvey
*Selbst-/fremdeingeschätzte Beeinträchtigung/Behinderung*	*13*	*30*	*80*	*85*	*5*	*11*	*13*	*103*	*3*	*61*	*14*	*39*	*151*	*42*	*260*
Arbeitssituation*	*58*	*250*	*244*	*62*	*55*	*119*	*102*	*12*	*7*	*183*	*109*	*117*	*46*	*216*	*57*
Stellung im Arbeitsmarkt	27	66	75	39	12	99	28	8	5	33	50	75	30	141	30
Unternehmensmerkmale	0	9	16	2	4	3	5	3	1	3	3	0	3	3	3
Betriebliche Rahmenbedingungen	13	15	9	2	5	8	15	0	0	48	21	13	0	17	11
Rahmenbedingungen der Tätigkeit	3	26	33	0	10	0	11	0	0	17	4	2	2	10	0
Arbeitszeitmerkmale	9	18	15	2	5	9	7	0	1	16	28	9	1	22	3
Arbeitsbedingungen	4	102	85	10	18	0	35	1	0	59	2	15	8	17	9
Arbeitszufriedenheit und Arbeitsmotivation	2	14	11	7	1	0	1	0	0	7	1	3	2	6	1
*Individuelle Merkmale*	*30*	*17*	*11*	*39*	*18*	*38*	*4*	*25*	*9*	*19*	*32*	*48*	*17*	*46*	*19*
*Gesamt*	*101*	*297*	*335*	*186*	*78*	*168*	*119*	*140*	*19*	*263*	*155*	*204*	*214*	*304*	*336*

* Die Anzahl der erfassten Items ergibt sich aus der Summe der nicht hervorgehobenen Unterkategorien

Die Arbeitssituation wird in den eingeschlossenen Befragungen in unterschiedlichem Maße abgedeckt. In Erwerbstätigenbefragungen (z. B. BIBB/BAuA, EWCS, lidA) sind tendenziell mehr Informationen zur Arbeitssituation zu finden. Es zeigt sich jedoch, dass beispielsweise EU-SILC und SOEP ebenfalls zahlreiche Informationen, insbesondere zur finanziellen Situation der Person/des Haushaltes (52 bzw. 50 Items) und zur Erwerbsbiografie (17 bzw. 32 Items; in der Kategorie „Stellung im Arbeitsmarkt“), beinhaltet. Je nach Fragestellung im Kontext der Arbeitssituation bieten sich unterschiedliche Befragungen an.

Hinsichtlich betrieblicher Rahmenbedingungen bietet zum Beispiel die lidA-Studie einen tiefergehenden Einblick in den Umgang mit behinderten/chronisch kranken Menschen anhand zahlreicher Informationen zur beruflichen Wiedereingliederung (35 Items). Darüber hinaus beinhaltet die lidA auch Informationen zum Thema „Arbeitszeiterfassung/-modell/-regelung“ (5 Items) oder zur „Vereinbarkeit Privatleben und Beruf“ (3 Items).

Rahmenbedingungen der Tätigkeit ebenso wie Arbeitsbedingungen sind besonders breit in der BIBB/BAuA und der BIBB/BAuA-Zusatz abgedeckt, insbesondere bei aktuellen Themen wie „Homeoffice/Telearbeit“ (7 bzw. 9 Items) und „Arbeit mit Technik/Werkzeugen/digitalen Medien“ (11 bzw. 22 Items). Des Weiteren werden hinsichtlich der Arbeitsbedingungen zahlreiche Belastungen in der BIBB/BAuA und BIBB/BAuA-Zusatz erhoben (102 bzw. 85 Items), darunter körperliche, umgebungsbezogene und psychosoziale Arbeitsbedingungen. Spezifisch für das Thema „barrierefreie Arbeitsbedingungen“ zeigen die lidA-Studie mit 14 Items, die BIBB/BAuA und der Teilhabesurvey mit jeweils 3 Items am meisten Analysepotenzial. Die anderen eingeschlossenen Befragungen (mit Ausnahme BIBB/BAuA-Zusatz – ein Item) haben keine Items zu diesem Thema integriert. Auch das Thema „Hilfsmittel im Arbeitskontext“ wird bislang eher wenig in bestehenden Befragungen erhoben. Hier bietet die BIBB/BAuA-Zusatz mit 9 Items den breitesten Blick auf Hilfsmittel. Die BIBB/BAuA und der Teilhabesurvey bieten Einblicke mit jeweils 3 Items.

Analysen von Arbeitszeitmerkmalen sind insbesondere mit dem MZ (28 Items) und dem SOEP (22 Items) möglich. Hier werden Themen wie „Arbeitszeitlänge“ (7 bzw. 6 Items), „Nacht‑/Schichtarbeit“ (MZ: 9 Items) oder „Überstunden“ (SOEP: 9 Items) erhoben.

Mit Ausnahme von EU-SILC, ISSP und GEDA bieten alle eingeschlossenen Befragungen die Möglichkeit, mindestens ein Item zu der Arbeitszufriedenheit oder -motivation auszuwerten. Insgesamt bietet die BIBB/BAuA mit 14 Items zur Arbeitszufriedenheit bzw. Zufriedenheit mit einzelnen arbeitsbezogenen Aspekten am meisten Analysemöglichkeiten. Die Arbeitsmotivation kann in der lidA-Befragung mit 3 Items und im EWCS mit einem Item analysiert werden.

Individuelle Merkmale sind in den Befragungen in einem ganz unterschiedlichen Maße abgedeckt. Dabei beinhalten aber alle Befragungen ein grundlegendes Set an Merkmalen wie Geschlecht, Alter, Bildung und Familienstatus. Zudem bieten zum Beispiel MZ, SOEP, DEAS, GEDA und PASS Informationen zum Migrationshintergrund bzw. zur Migrationserfahrung. Aber auch Items zum Thema „soziale Unterstützung im privaten Umfeld“ liegen zum Beispiel in den Befragungen AID:A, DEAS oder EU-SILC vor (Tabelle Z2 im Onlinematerial).

Ferner wird deutlich, dass bestimmte Kategorien, die einen ganzheitlicheren Blick auf die Arbeitssituation von Menschen mit Beeinträchtigung bzw. Behinderung geben könnten, bislang kaum oder auch gar nicht in den Befragungen abgedeckt sind. Im Kontext der Unternehmensmerkmale zeigt sich, dass Informationen zur wirtschaftlichen Lage des Betriebs bislang kaum vorliegen. So ist diese Information momentan nur in der BIBB/BAUA-Zusatz enthalten. In der Hauptkategorie betriebliche Rahmenbedingungen sind Informationen zu den Unterkategorien „Personal‑/Betriebsrat/Gewerkschaft“, „Beauftragte/r für Arbeitsschutz und Gesundheit“ oder „Unternehmenskultur/Betriebsklima“ ebenfalls nur vereinzelt zu finden (z. B. BIBB/BAuA, EWCS, Teilhabesurvey). Informationen darüber, ob ein/e Inklusionsbeauftragte/r vorhanden ist, ob das Unternehmen soziale Verantwortung übernimmt und ob Personalengpässe vorliegen, sind gar nicht vorhanden. In der Hauptkategorie Rahmenbedingungen der Tätigkeiten sind neue Steuerungsformen wie „Ergebnisorientierung/Eigenverantwortung“, „Zielvereinbarungen“ und „Leistungssteuerung/-kontrolle“ gar nicht bzw. nur teilweise mit jeweils einem Item in der BIBB/BAuA abgedeckt. Im Kontext der Arbeitszeitmerkmale beinhaltet gerade die Unterkategorie „Pausen (z. B. Einfluss, Ausfall)“ kaum Items. So bieten bezüglich Pausen jeweils die BIBB/BAuA und die BIBB/BAuA-Zusatz ein Item. Neben den bereits genannten Unterkategorien „barrierefreie Arbeitsbedingungen“ und „Hilfsmittel im Arbeitskontext“ werden weitere Aspekte aus der Hauptkategorie Arbeitsbedingungen kaum bis gar nicht abgedeckt, darunter „wiederholte Bewegungsabläufe“, „emotionale Anforderungen“ und „arbeitsbezogene Konflikte“. Zudem ist der Bereich „übergriffiges Verhalten/Belästigung/Diskriminierung“ eher selten abgedeckt. Möglichkeiten zur Analyse bieten hier lediglich EWCS (4 Items), MZ (2 Items) und lidA (1 Item; Tabelle Z2 im Onlinematerial).

## Diskussion

Die vorliegende systematische Übersicht von Großbefragungen in Deutschland zeigt, dass einige Befragungen bereits Teilaspekte der Arbeitssituation von Menschen mit Beeinträchtigung bzw. Behinderung auf dem ersten Arbeitsmarkt abbilden. Damit bieten diese einen weiteren Baustein in der Betrachtung der Arbeitssituation von Menschen mit Beeinträchtigung neben Erhebungen bzw. Studien, die aufgrund der gewählten Einschlusskriterien nicht Teil der Übersichtsarbeit sind.

Für 2018–2024 wurden 7 Befragungen (BIBB/BAuA, BIBB/BAuA-Zusatz, DEAS, lidA, PASS, SOEP und Teilhabesurvey) identifiziert, in denen die 3 Zielgruppen Menschen mit Beeinträchtigung und mit selbst- sowie fremdeingeschätzter Behinderung unterschieden werden können und in denen Informationen zur Arbeitssituation sowie zu individuellen Merkmalen erhoben werden. Acht weitere Befragungen decken zumindest einen Teil der genannten Zielgruppen sowie Merkmale der Arbeitssituation ab.

Neben dem bereits bestehenden Analysepotenzial der betrachteten Befragungen wird darüber hinaus deutlich, dass es bislang an einer Großbefragung fehlt, die im Sinne einer teilhabeorientierten Erwerbstätigenbefragung umfänglich den Themenbereich „Beeinträchtigung/Behinderung“ und den Bereich „Arbeitssituation“ mit den verschiedenen Teilbereichen abdeckt. So bieten GEDA, SHARE und insbesondere der Teilhabesurvey umfängliche Frageprogramme, um die Themen „Beeinträchtigung/Behinderung“ abzudecken. Dementgegen zeigt sich in den Erwerbstätigenbefragungen, die einen Schwerpunkt auf dem Thema „Arbeit“ haben, wie zum Beispiel BIBB/BAuA, EWCS oder lidA, noch Ausbaupotenzial. Dies betrifft unter anderem Informationen zu Art der Beeinträchtigung, Hilfsmittelnutzung und Arbeitseinschränkungen. Insgesamt wird deutlich, dass zentrale Anforderungen, etwa die Abbildung der ICF-basierten Definition von Behinderung, die Anschlussfähigkeit an sozialrechtliche Definitionen sowie die ganzheitliche Betrachtung von arbeitsbezogenen Kontextfaktoren, die komplexe Teilhabebedingungen prägen, bislang nur in Teilen erfüllt sind. Daraus ergeben sich Weiterentwicklungspotenziale für zukünftige Befragungen.

### Erhebungskonzepte von Beeinträchtigung und Behinderung

Nach dem biopsychosozialen Modell der ICF entsteht Behinderung aus der Interaktion von Gesundheitsproblemen mit personenbezogenen und umweltbezogenen Kontextfaktoren [26]. Diese Perspektive wird in den meisten Erhebungen bislang nicht systematisch umgesetzt. Eine Ausnahme ist der Teilhabesurvey, der sowohl Informationen zu gesundheitlichen Einschränkungen, funktionalen Beeinträchtigungen und Umweltfaktoren (z. B. Barrieren, Unterstützung) erhebt. Diese Daten erlauben eine ICF-nahe Abbildung, insbesondere durch die Erfassung von Aktivitäts- und Teilhabebeschränkungen sowie Kontextmerkmalen wie der Arbeitsumgebung. Zusätzlich bieten BIBB/BAuA, BIBB/BAuA-Zusatz, DEAS, lidA, PASS und SOEP Möglichkeiten, Beeinträchtigung und Behinderung zu differenzieren. Eine kritische Analyse des Messkonzepts des Teilhabesurveys durch Schäfers zeigt allerdings, dass sich dieses nicht konsistent an der ICF orientiert [30]. Kritikpunkte sind unter anderem die unklare Definition von Beeinträchtigung und der geringe Stellenwert von Umweltfaktoren als Ursache für eine Behinderung. Ferner erfolgt eine Abgrenzung von Beeinträchtigung und Behinderung ohne eine stimmige Begründung. Schäfers schlägt wie bereits Coenen et al. [31] vor, den WHO Model Disability Survey (MDS) zur Messung von Beeinträchtigung und Behinderung zu nutzen [30]. Mit dem MDS können Daten über alle Dimensionen von Behinderung erfasst werden, wie Informationen über Beeinträchtigungen, Aktivitätseinschränkungen, Teilhabebeschränkungen sowie über Umweltfaktoren, die die vollständige Teilhabe entweder erleichtern oder behindern [32]. Da nicht in jede Befragung die Integration des MDS möglich (und auch sinnvoll) ist, kann die Gruppeneinteilung ausschließlich nach der Beeinträchtigungsstärke unter Verzicht auf die grundsätzliche Unterscheidung zwischen Beeinträchtigung und Behinderung ein alternatives Messkonzept darstellen [30].

### Indikatoren zur Arbeitssituation

Einzelne relevante Aspekte zur Beschreibung der Arbeitssituation finden bislang nur kaum oder gar keine Berücksichtigung. Neben Themen, die allgemein in der Arbeitswelt von Bedeutung sind und Auswirkungen auf das Wohlbefinden aller Beschäftigten haben können, wie zum Beispiel Personalengpässe, emotionale Anforderungen oder neue Steuerungsformen wie Eigenverantwortung bei der Arbeit [33–36], zeigen sich Lücken, die insbesondere im Kontext der beruflichen Teilhabe von Belang sind.

Mit Blick auf die betrieblichen Rahmenbedingungen liegen in den untersuchten Befragungen keine oder nur wenige Informationen vor, mit denen strukturelle Rahmenbedingungen oder kulturelle Aspekte in einer Organisation beleuchtet werden könnten, beispielsweise das Vorhandensein von Inklusionsbeauftragten, eines inklusiven Betriebsklimas oder gezielter Unterstützungsmaßnahmen am Arbeitsplatz für Menschen mit Beeinträchtigung/Behinderung. Dabei stellen die betrieblichen Rahmenbedingungen einen zentralen Faktor für die berufliche Inklusion dar [29, 37–39].

Ferner ist aktuell die Informationsgrundlage zur Barrierefreiheit von Arbeitsbedingungen sowie zur Nutzung bzw. Bedarfsdeckung von Hilfsmitteln bei der Arbeit und damit das zentrale Thema „Arbeitsplatzanpassungen“ [13, 40, 41] weiter auszubauen. Nur wenige Erhebungen (lidA, Teilhabesurvey, BIBB/BAuA(‑Zusatz)) erfassen explizit barrierefreie Arbeitsbedingungen oder den Einsatz von Hilfsmitteln am Arbeitsplatz. Dabei beziehen sich die Informationen in Teilen auf spezifische Kontexte (z. B. Hilfsmittel im Kontext von Rehabilitationsmaßnahmen – lidA) oder sind nicht nachhaltig angelegt, da es sich um einmalige Befragungen handelt (BIBB/BAuA-Zusatz). Eine systematische Erhebung dieser Aspekte würde es ermöglichen, Versorgungs- und Schutzlücken zu identifizieren und gezielt politische oder betriebliche Maßnahmen zu initiieren.

### Inklusive Studiendesigns

Nicht zuletzt spielt das Erhebungsdesign eine zentrale Rolle, um die diverse Gruppe von Menschen mit Beeinträchtigung bzw. Behinderung und deren Arbeitssituationen abbilden zu können. Der Teilhabesurvey, der den Anspruch einer inklusiven Befragung hat, zeigt auf, dass es eines mehrstufigen Stichprobendesigns bedarf, um Personen mit Beeinträchtigungen und selbsteingeschätzten Behinderungen angemessen zu ermitteln [42]. Ebenfalls müssen verschiedene Erhebungsmethoden zum Einsatz kommen, um allen Personen unabhängig von der Art und Schwere einer Beeinträchtigung die Teilhabe an einer Befragung zu ermöglichen [43]. Nicht alle Befragungen sind darauf ausgelegt, diese Anforderungen zu erfüllen. Allerdings sollte bei der (Weiter‑)Entwicklung von Befragungen mitgedacht werden, über welche Erhebungsmethoden unterrepräsentierte Gruppen besser einbezogen werden können.

### Ausbau der Befragungslandschaft

Die UN-Behindertenrechtskonvention verpflichtet Deutschland, Daten zur Verwirklichung der Rechte von Menschen mit Behinderung systematisch zu erheben (Art. 31 UN-BRK; [16]). Die Ergebnisse dieser Übersicht zeigen, dass die derzeitige Datengrundlage basierend auf Großbefragungen eine erste, aber keine hinreichende Grundlage für ein Monitoring der Umsetzung im Teilhabefeld „Arbeit und Beschäftigung“ bietet. So fehlt eine durchgängige teilhabeorientierte Operationalisierung von Behinderung, wie sie durch die WHO oder das Teilhabekonzept der Bundesregierung intendiert ist. Auch werden zentrale Aspekte des Rechts auf Arbeit (Art. 27 UN-BRK; [16]) – etwa gleichwertige Arbeitsbedingungen, barrierefreie Gestaltung oder Schutz vor Diskriminierung – nur unzureichend erfasst. Eine konsequente Umsetzung der UN-BRK erfordert daher den Ausbau der Befragungslandschaft – sowohl hinsichtlich Erhebungsdesign (barrierefreie Zugänge, differenzierte Stichproben) als auch hinsichtlich der inhaltlichen Abbildung (z. B. zu Arbeitsplatzanpassung, inklusiver Betriebskultur oder Diskriminierungserfahrungen). Für den politischen Diskurs bedeutet das: Aussagen zu Ungleichheiten im Arbeitsleben sind möglich, die Wirksamkeit von Inklusionspolitik ist jedoch schwer evaluierbar.

Gleichermaßen bleiben Ansatzpunkte für eine gesundheitsförderliche Arbeitsgestaltung von Menschen mit Beeinträchtigung bzw. Behinderung unentdeckt. Insbesondere fehlen kontinuierliche, indikatorengestützte Daten zur Barrierefreiheit am Arbeitsplatz und zu betrieblichen (Inklusions‑)Strategien. Bestehende Großerhebungen müssten daher systematisch erweitert werden – inhaltlich durch differenzierte Items und methodisch durch inklusive Stichproben- und Erhebungsdesigns. Das von infas (Institut für angewandte Sozialwissenschaft GmbH) und der BAuA entwickelte Befragungskonzept im Rahmen der Pilotstudie „Förderung der Barrierefreiheit bei der Datenerhebung von Arbeitsbedingungen“ bietet hierfür wichtige Impulse (Abschlussbericht, inkl. entwickelten Fragebogens, siehe [44]). Es verbindet ein inklusives Stichprobendesign mit einem Mixed-Mode-Design und einem umfassenden Fragebogen, der neben Aspekten, die allgemein die Qualität der Arbeit beschreiben, auch inklusionsspezifische Inhalte abgedeckt.

## Fazit

Die systematische Übersicht zeigt, dass bestehende Großbefragungen in Deutschland bereits Teilaspekte der Arbeitssituation von Menschen mit Beeinträchtigung oder Behinderung erfassen, jedoch keine umfassende teilhabeorientierte Datengrundlage bieten. Insbesondere fehlen Erhebungen, die Beeinträchtigung/Behinderung im Sinne des biopsychosozialen Modells operationalisieren und sowohl individuelle als auch arbeitsbezogene Kontextfaktoren berücksichtigen. Dies führt zu einem fragmentierten Blick auf die Arbeitssituation von Menschen mit Beeinträchtigung/Behinderung in Deutschland, bei dem zentrale Aspekte von Teilhabe wie betriebliche Inklusionskultur, Barrierefreiheit oder Arbeitsplatzanpassungen unterbelichtet bleiben. Inhaltliche Weiterentwicklungsmöglichkeiten für Befragungen liegen insbesondere in einer konsequent teilhabeorientierten Operationalisierung von Behinderung und der systematischen Berücksichtigung von Indikatoren zu Barrierefreiheit, Hilfsmitteln, inklusiver Unternehmenskultur und Diskriminierung. Methodische Verbesserungspotenziale liegen in der Umsetzung inklusiver Befragungskonzepte, die sich insbesondere durch eine erhöhte Flexibilität bei Erhebungsstrategien auszeichnen. Eine teilhabeorientierte Weiterentwicklung der Befragungslandschaft könnte neue Datenpotenziale eröffnen, anhand derer evidenzbasierte Strategien zur Förderung inklusiver Arbeitsbedingungen entwickelt und evaluiert werden können.

## Supplementary Information

ESM1: Zusatzmaterial 1

## Footnotes

1 Einen Überblick über Art, Zugang, Qualität und datenschutzrechtliche Aspekte zu Routinedaten im Kontext der Rehabilitationsforschung geben Brünger et al. [17].
